# Preterm birth and nativity among Black women with gestational diabetes in California, 2013–2017: a population-based retrospective cohort study

**DOI:** 10.1186/s12884-020-03290-3

**Published:** 2020-10-06

**Authors:** Karen A. Scott, Brittany D. Chambers, Rebecca J. Baer, Kelli K. Ryckman, Monica R. McLemore, Laura L. Jelliffe-Pawlowski

**Affiliations:** 1 Department of Obstetrics, Gynecology & Reproductive Sciences, School of Medicine, University of California San Francisco, 2356 Sutter Street, J-140, San Francisco, CA 94143 USA; 2 California Preterm Birth Initiative, University of California San Francisco, 3333 California Street, Suite 285, San Francisco, CA 94118 USA; 3 Department of Epidemiology and Biostatistics, School of Medicine, University of California San Francisco, 560 16th Street, Second Floor, San Francisco, CA 94158 USA; 4 Department of Pediatrics, University of California San Diego, 9500 Gilman Drive, Building 3, La Jolla, CA 92161 USA; 5 Department of Epidemiology, University of Iowa College of Public Health, 145 N. Riverside Drive, Office S435 CPHB, Iowa City, IA 52242 USA; 6 Family Health Care Nursing Department, School of Nursing, University of California, 2 Koret Way, N431H, San Francisco, San Francisco, CA 94143 USA

**Keywords:** Black women, Preterm birth severity, Preterm birth subtypes, Gestational diabetes, Nativity, Preeclampsia, Anti-racist praxis

## Abstract

**Background:**

Despite the disproportionate prevalence of gestational diabetes (GDM) and preterm birth (PTB) and their associated adverse perinatal outcomes among Black women, little is known about PTB among Black women with GDM. Specifically, the relationship between PTB by subtype (defined as indicated PTB and spontaneous PT labor) and severity, GDM, and nativity has not been well characterized. Here we examine the risk of PTB by severity (early < 34 weeks, late 34 to 36 weeks) and early term birth (37 to 38 weeks) by nativity among Black women with GDM in California.

**Methods:**

This retrospective cohort study used linked birth certificate and hospital discharge data for 8609 of the 100,691 self-identifying non-Hispanic Black women with GDM who had a singleton live birth between 20 and 44 weeks gestation in California in 2013–2017. Adjusted odds ratios (aOR) and 95% confidence intervals (CIs) were examine risks for PTB, by severity and subtype, and early term birth using multivariate regression modeling.

**Results:**

Approximately, 83.9% of Black women with GDM were US-born and 16.1% were foreign-born. The overall prevalence of early PTB, late PTB, and early term birth was 3.8, 9.5, and 29.9%, respectively. Excluding history of prior PTB, preeclampsia was the greatest overall risk factor for early PTB (cOR = 6.7, 95%, CI 5.3 to 8.3), late PTB (cOR = 4.3, 95%, CI 3.8 to 5.0), and early term birth (cOR = 1.8, 95%, CI 1.6 to 2.0). There was no significant difference in the prevalence of PTB by subtypes and nativity (*p* = 0.5963). Overall, 14.2% of US- compared to 8.9% of foreign-born women had a PTB (early PTB: aOR = 0.56, 95%, CI 0.38 to 0.82; late PTB: aOR = 0.57, 95%, CI 0.45 to 0.73; early term birth: aOR = 0.67, 95%, CI 0.58 to 0.77).

**Conclusions:**

Foreign-born status remained protective of PTB, irrespective of severity and subtype. Preeclampsia, PTB, and GDM share pathophysiologic mechanisms suggesting a need to better understand differences in perinatal stress, chronic disease, and vascular dysfunction based on nativity in future epidemiologic studies and health services research.

## Background

Nearly 86% of pregnancies impacted by diabetes are the result of gestational diabetes mellitus (GDM) [1, 2]. GDM, like preterm birth (PTB, < 37 weeks), disproportionately develops in the pregnancies of Black women [3] and significantly contributes to both maternal and infant mortality and morbidity [4–6]. However, PTB outcomes in pregnancies affected by GDM remains unexplained in published literature, particularly in Black women. Risk of PTB and GDM among Black women differs by one notable risk factor, namely maternal country of origin or nativity. Foreign-born Black women are less likely than Black women born in the United States (U.S.) to experience PTB [7–10], although they may be more likely to develop GDM [11–13]. The protective effect of foreign birth outside of the U.S. has been attributed to a phenomenon called the healthy migrant theory [14], which describes how foreign-born persons who migrate to the U.S. have significantly better health outcomes in comparison to U.S.-born persons with the same racial and ethnic identities [15–19].

In the U.S., maternal country of origin may be a proxy for the impact of structural oppressions and life course experiences of over/chronic exposure to gendered racism, classism, and other forms of discrimination unique to Black women. However, traditional “big data” epidemiological studies that rely on administrative databases tend not to contain structural and neighborhood factors to allow for temporal or spatial contextualization of the data beyond individual behavior or characteristics. Thus, we propose the application of a public health critical race (PHCR) praxis as a methodology to interrogate the racialized social and clinical phenomenon of PTB among Black women with GDM in California in quantitative population health research. In this manner, we hope to avoid the reproduction of “mother blame” narratives [20] and structural racism [21] in our assumptions and approaches to both understanding and describing individual-level factors associated with differences in PTB among Black women whose pregnancies were affected by GDM and other clinical events.

To date, there are limited data that describe the association of nativity and PTB by severity and subtype, and early term birth, respectively, in a high-risk population of Black women with GDM in California. To address this gap, we examined the risk of early PTB (< 34 weeks), late PTB (34–36 weeks), and early term birth (37–38 weeks) by nativity among Black women with GDM.

### Anti-racist praxis, study assumptions, and methodologies

The utilization of the PHCR praxis informs two key study assumptions: 1) structural racism creates a hierarchy of power, domination, and privilege in society that impacts the distribution and expression of illness and health as well as access to and utilization of resources to meet and maintain basic social needs (i.e., food, housing, water, family/community stability, employment, and safety [22]); and 2) the structural context of living as a Black woman in the U.S. impacts perinatal and reproductive health (PRH) and health care outcomes and experiences in a manner not reported nor described in white women [23–27] or foreign-born Black women. The implications of the PHCR praxis inform the methodologies in four focuses as defined by Ford and Airhihenbuwa [28]: 1) Contemporary Patterns of Racial Relations: We would like to situate the racialized clinical and social phenomenon of PTB in the U.S. within both a temporal and spatial context, underscoring the ongoing threats to achieving and advancing PRH equity, based on four trends: a) Feminization of the workforce without a concomitant equitable racial/ethnic diversification of the workforce; b) geographic maldistribution of the workforce and services in rural areas, urban areas, and areas adjacent to urban centers; c) absence of any OBGYN physicians in nearly half of U.S. counties due to the shortage or absence of a hospital with obstetric care and/or an obstetric provider (i.e., maternal care desert); and d) lack of racial concordance between the workforce and the public, particularly in rural areas, urban areas, and areas adjacent to urban centers in the Midwest and Southern regions [29]; 2) Knowledge Production: Existing evidence that posits PTB as an intrinsic biological defect in Blackness and Black birthing bodies [30–33] needs to be deeply interrogated. A group of Black and Brown women scholars in obstetrics gynecology, maternal fetal medicine, family planning, and public health recently asserted that “despite evidence describing the relationship between structural racism, health outcomes and healthcare care experiences in the literature by transdisciplinary experts in the social sciences, humanities, legal studies, public health, and health services research, knowledge construction by obstetricians, perinatologists, and gynecologists about the impact of structural racism on PRH inequity is lacking” [29]. Quantitative public health research on PRH, specifically PTB, by Black women scholars provide substantial evidence, with both scientific and cultural rigor, on the utility of correlating adverse PRH outcomes among Black birthing communities with traditional and novel measures of structural racism, such as neighborhood level segregation and census tract data [34–37], and county-level disparities in incarceration and elected officials [38]; 3) Conceptualization and Measurement: The exclusion of white women as a control group for all three gestational groups (early PTB, late PTB, and early term) allows for better examination of within group variations among Black women birthing in California hospitals based on nativity [8, 39] and other clinical factors without reinforcing race as a biological construct or advantage in examining the racialized phenomenon of pregnancy, GDM, and PTB among Black women [40]. Likewise, utilization of interaction analysis and backward stepwise regression modeling allows for the operationalization of intersectionality in quantitative population health research [18, 41–43]. We started with a fully saturated model that included nativity, gestational groups, covariates, confounders, and interaction terms, allowing for the exploration of the simultaneous joint effects of nativity – covariate and covariate-covariate on PTB and early term birth, beyond the explanatory paradigm of the sum of the individual effect of nativity or maternal and obstetric characteristics; and 4) Action: We use knowledge generated from our study to disrupt the reliance of clinical outcomes as defined in administrative databases as the predominant approach to examining the causes of PTB inequities. Instead, we propose the use of a patient reported outcome or experience measure of PTB among Black women with GDM in clinical settings, particularly when clinical significance outweighs statistical significance [44].

## Methods

### Study population

**Fig. 1:**
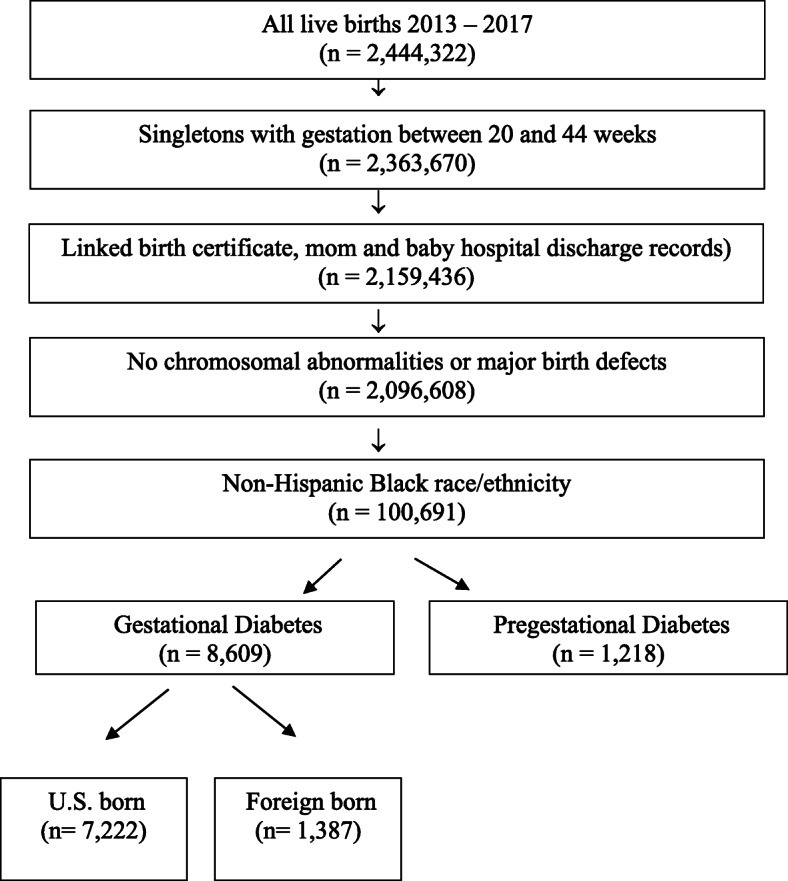
Sample selection

This retrospective cohort study used linked birth certificate and hospital discharge data for 2,096,608 women who had live singleton births between 20 and 44 weeks gestation without chromosomal abnormalities or major birth defects in California from 2013 to 2017. From a population of 100,691 self-identifying Black women in this sample, we identified 8609 non-Hispanic Black women with GDM for this study. Fig. 1 illustrates the sample selection process.

Birth certificates, maintained by California Vital Statistics, were linked to a hospital discharge, emergency department, and ambulatory surgery records maintained by the California Office of Statewide Health Planning and Development. These databases contain detailed information on maternal and infant characteristics, hospital discharge diagnoses, and procedures. Hospital discharge, emergency department, and ambulatory surgery files provided diagnoses and procedure codes based on the International Classification of Diseases, 9th Revision, Clinical Modification (ICD-9) and International Classification of Diseases, 10th Revision, Clinical Modification (ICD-10) as reported to the California Office of Statewide Health Planning and Development by the health care facilities.

### Measures

Outcome variables were organized into four gestational groups: < 34 weeks (cases, early PTB), between 34 and 36 weeks (cases, late PTB), between 37 0/7 to 38 6/7 weeks (cases, early term birth), and term birth [> 39 to 44 weeks, controls, inclusive of full term (39 to 40 weeks), late term (41 to < 41 weeks), and post-term (42 weeks and beyond)]. Best obstetric estimate of gestation at birth was obtained from birth certificate records [45]. PTB was evaluated by subtype [46]. The predictor variable was defined as the self-defined maternal country of birth (hereinafter referred to as nativity). Covariate variables included maternal age at birth (< 24 years old, 24–39 years old, > 40 years old), history of PTB among multiparous birthing persons (yes/no), maternal education (< 12 years, 12 years, and > 12 years), participation in women, infant, and children’s (WIC) program (yes/no), diagnosis of preeclampsia in the index pregnancy (yes/no), smoking during pregnancy (yes/no), payer status for birth (employer health/individual plans, public insurance or Medi-Cal (California’s Medicaid program or state funded health insurance for low income individuals), self-pay, or other employer based/individual insurance plans), pre-pregnancy body mass index (BMI = weight in kilograms divided by height in meters^2^) across six categories (< 18.5 (underweight); 18.5 to 24.9 (normal); 24–29.9 (overweight); > 30 kg/m^2^ (obese); obesity defined as BMI > 30 across 3 categories (BMI of 30 to < 35 (Class 1); BMI of 35 to < 40 (Class 2); BMI of 40 of higher (Class 3, also known as “extreme” or “severe” obesity)), adequacy of prenatal care utilization (PNC, adequate/adequate plus, intermediate, inadequate per Kotelchuck [47], and birth type (vaginal birth, cesarean birth). Data were extracted from birth certificates and hospital discharge records using the International Classification of Disease (ICD), 9th Revision, and ICD 10th Revision Clinical Modification [48, 49] for GDM (648.8) and preeclampsia (642.4–7) [50].

Three types of PTB were assessed: preterm premature rupture of membranes (PPROM, defined a rupture of membranes prior to onset of labor before 37 gestational weeks), spontaneous labor with intact membranes, and provider initiated for maternal, placental, or fetal indications. The birth was identified as spontaneous PTB when medical records demonstrated PPROM or spontaneous labor with intact membranes. PTB was determined by gestational age at birth. As previously described by Jelliffe-Pawlowski et al. [51] in 2015, women whose birth certificate or hospital discharge records noted preterm labor or tocolytic medication but had no indication of PPROM were included in the spontaneous preterm labor with intact membranes group. All pregnancies with PPROM noted in the infant’s birth certificate or mother’s hospital discharge records were included in the PPROM group. Pregnancies without PPROM, preterm labor, or tocolytic administration for which there was a code for “medical induction, “surgical induction” or “artificial rupture of membrane”; or for which there was a cesarean birth at < 37 weeks of gestational without any of the aforementioned codes were included in the provider-initiated birth group. Pregnancies without indication of spontaneous or provided initiated PTB were categorized as “unknown”.

### Statistical methods

Univariate and bivariate comparisons of gestational groups were determined using chi-square analysis between U.S.-born (the referent group) and foreign-born Black women. Bivariate logistic regression was used to calculate the crude odds ratios (cORs) and 95% confidence intervals (CIs) for the associations of nativity and gestational duration adjusted individually by covariates. In addition, odds ratios, adjusted for maternal and obstetric characteristics (aORs) and 95% CIs were calculated to evaluate the relative risk of PTB, by severity and subtype, and early term birth using multivariate regression modeling.

Effect modification was determined by calculating stratum-specific estimates of associations between covariates and each gestational group using the Breslow-Day test for homogeneity of the risk ratios with statistical significance, *p* <  0.05. To assess for confounding, the cORs, aORs, and standard Wald 95% CIs were examined for differences in gestational outcome groups by these covariates based on nativity. If the magnitude of adjusted association changed by 10% or greater, the potential confounder was included in the final model.

Backward stepwise regression was used for final model building (using *p* < 0.10 to enter the model and *p* < 0.05 to remain in the model), with inclusion of covariates and interaction terms between covariates and nativity with a significant association with each the three gestational outcome groups as well as confounders between nativity and the gestational groups. Likewise, interaction terms between covariates were assessed that were considered clinically important based on the clinician authors domain knowledge and areas of expertise in high risk obstetrics hospitalist care, public health, and applied epidemiology, and found to be relevant in the literature and clinical practice across all three gestational groups.

Statistical Analysis Software version 9.4 (Cary, NC) was used to analyze data received by the California Preterm Birth Initiative at the University of California San Francisco as of March 1, 2020. Methods and protocols for the study were approved by the Committee for the Protection of Human Subjects within the Health and Human Services Agency of the State of California.

## Results

Demographic characteristics of study participants are shown in Table 1. Most (*n* = 7222 of 8609; 83.89%) U.S.-born Black women with GDM, compared to foreign-born, were more likely to give birth under the age of 25 years (*n* = 1014, 14.0%), obtain 12 or fewer years of formal education (*n* = 2915, 40.4%%), participate in WIC (*n* = 4785, 66.3%), develop preeclampsia at birth (*n* = 971, 13.5%), smoke in pregnancy (*n* = 725, 10.0%), use Medi-Cal (*n* = 4024, 55.7%), have pre-pregnancy obesity class I (*n* = 1539, 49.6%), class II (*n* = 1168, 16.2%), and class III (*n* = 1245, *n* = 17.2%), and achieve adequate or adequate plus prenatal care (*n* = 5357, 74,3%) (Table 1). History of birth type demonstrated no significant association with nativity (Table 1). The prevalence of a prior PTB among Black women with GDM was 2.9% (*n* = 245).

**Table 1:** Distribution of maternal and obstetric sample characteristics by nativity, 2013–2017, *N* = 8609

	U.S.-born *n* = 7222	Foreign-born *n* = 1387	Total *n* = 8609	*p* value (by χ^2^)
	n (%)	n (%)	n (%)	
Maternal Age (years)				**< 0.0001**
< 24	1014 (14.0)	30 (2.2)	1044 (12.1)	
24–39	5751 (79.6)	1187 (85.6)	6938 (80.6)	
> 40	457 (6.3)	170 (12.3)	627 (7.3)	
Prior PTB				0.0033
Yes	222 (3.1)	23 (1.7)	245 (2.9)	
No	6991 (96.9)	1373 (98.4)	8364 (97.2)	
Parity				0.6771
Nulliparous	2130 (29.5)	420 (30.1)	2550 (29.8)	
Multiparous/missing parity	5083 (70.5)	976 (69.9)	6059 (70.4)	
Education				**< 0.0001**
< 12	704 (9.8)	88 (6.3)	792 (9.2)	
12	2211 (30.6)	297 (21.4)	2508 (29.1)	
> 12	4099 (56.8)	926 (66.8)	5025 (58.4)	
Missing values	208 (2.9)	76 (5.5)	284 (3.3)	
Participation in WIC				**< 0.0001**
Yes	4785 (66.3)	715 (51.6)	5500 (63.9)	
No	2397 (33.2)	663 (47.8)	3060 (35.5)	
Missing values	40 (0.6)	9 (0.7)	49 (0.6)	
Preeclampsia				**0.0014**
Yes	971 (13.5)	143 (10.3)	1114 (12.9)	
No	6251 (86.6)	1244 (89.7)	7495 (87.1)	
Smoking during pregnancy				**< 0.0001**
Yes	725 (10.0)	8 (0.6)	733 (8.5)	
No	6497 (90.0)	1379 (99.4)	7876 (91.5)	
Payer status for birth				**< 0.0001**
Employer health/individual plans	2715 (37.6)	601 (43.3)	3316 (38.5)	
Medi-Cal/Public insurance	4024 (55.7)	687 (49.5)	4711 (54.7)	
Self-Pay	49 (0.7)	31 (2.2)	80 (0.9)	
Other	434 (6.0)	68 (4.9)	502 (5.8)	
Body mass index [BMI (kg/m^2^)]				**< 0.0001**
< 18.5	93 (1.3)	29 (2.1)	122 (1.4)	
18.5 to < 25	1235 (17.1)	434 (31.3)	1669 (19.4)	
25 to < 30	1561 (21.6)	437 (31.5)	1998 (23.2)	
30 to < 35	1539 (21.3)	262 (18.9)	1801 (20.9)	
35 to < 40	1168 (16.2)	85 (6.1)	1253 (14.6)	
> 40	1245 (17.2)	57 (4.1)	1302 (15.1)	
Missing values	381 (5.3)	83 (6.0)	464 (5.4)	
Adequacy of prenatal care				**0.0032**
Adequate/adequate plus	5367 (74.3)	965 (69.6)	6332 (73.6)	
Intermediate	854 (11.8)	193 (13.9)	1047 (12.2)	
Inadequate	797 (11.0)	186 (13.4)	983 (11.4)	
Missing	204 (2.8)	43 (3.1)	247 (2.9)	
Birth Type				0.8498
Vaginal Birth	3630 (50.3)	701 (50.5)	4331 (50.3)	
Cesarean Birth	3592 (49.7)	686 (49.5)	4278 (49.7)	

^a^not presented when *n* < 5

### Outcome variables – early PTB

There were 328 (3.8%) early PTBs, 820 (9.5%) late PTBs, 2570 (29.9%) early term births, and 4891 (56.8%) full term births in the sample. Compared with U.S.-born Black women with GDM, the odds of early PTB, late PTB and early term birth were reduced by 50, 40, and 30% respectively (cOR 0.5 (95% CI 0.40, 0.80), cOR 0.6 (0.69, 0.85), and cOR 0.7 (0.7, 0.8) (Table 2) among foreign-born Black women with GDM. Maternal age < 24 (cOR 0.7 (0.5, 1.0)) and intermediate PNC utilization (cOR 0.6 (0.4, 0.8)) were significantly associated with a lower odds of early PTB. Prior PTB (cOR 7.3 (4.8–11.2), preeclampsia (cOR 6.7 (5.3, 8.3)), smoking (cOR 1.7 (1.2, 2.3)), and class II obesity (cOR 1.6 (1.1, 2.3)) were significantly associated with increased odds of early PTB among Black women with GDM. No significant associations were observed between early PTB and education, WIC participation, and payer status at birth (Table 2). Confounders between nativity and early PTB included smoking, preeclampsia, and pre-pregnancy BMI. Significant interactions between nativity and early PTB, and between maternal characteristics, included WIC participation*nativity (*p* = 0.0293), preeclampsia*smoking (*p* = 0.0008), and smoking*obesity (*p* = 0.0004) (Table 3).

**Table 2:** Comparison of maternal, social, and obstetric characteristics by gestational groups for Black mothers with GDM, *N* = 8609

	< 34 weeks *n* = 328	34–36 weeks *n* = 820	37–38 weeks *n* = 2570	≥39 weeks *n* = 4891
	n (%)	n (%)	n (%)	n (%)
	cOR (95% CI)	cOR (95% CI)	cOR (95% CI)	cOR (95% CI)
	*p* value	*p* value	*p* value	*p* value
**Nativity**				
US born	292 (89.0)	732 (89.3)	2236 (87.0)	3962 (81.0)
				Reference
Foreign-Born	36 (11.0)	88 (10.7)	334 (13.0)	929 (19.0)
	0.5 (0.4, 0.8)	0.6 (0.4, 0.7)	0.7 (0.7, 0.8)	
	**0.0006**	**< 0.0001**	**< 0.0001**	
**Maternal Age (years)**				
< 24	28 (8.5)	96 (11.7)	312 (12.1)	608 (12.4)
	0.7 (0.5, 1.0)	0.9 (0.8, 1.2)	1.0 (0.9, 1.1)	
	**0.0442**	0.6070	0.7720	
24–39	300 (91.5)	721 (87.9)	2255 (87.7)	4279 (87.5)
				Reference
> 40	a	a	a	a
**Education (years)**				
< 12	33 (10.1)	108 (13.2)	212 (8.3)	439 (9.0)
	1.3 (0.8, 1.9)	1.4 (1.1, 1.7)	1.0 (0.8, 1.1)	
	0.2491	**0.0041**	0.7607	
12	85 (25.9)	240 (29.3)	728 (28.3)	1455 (29.8)
				Reference
> 12	202 (61.6)	449 (54.8)	1550 (60.3)	2824 (57.7)
	1.2 (0.9, 1.6)	1.0 (0.8, 1.1)	1.1 (1.0, 1.2)	
	0.1413	0.6923	0.1765	
**Participation in WIC**				
Yes	194 (59.2)	548 (66.8)	1632 (63.5)	3126 (63.9)
	0.8 (0.7, 1.0)	1.1 (1.0, 1.3)	1.0 (0.9, 1.1)	
	0.1077	0.1395	0.7858	
No	131 (39.9)	268 (32.7)	923 (35.9)	1738 (35.5)
				Reference
**Adequacy of prenatal care**				
Adequate/adequate plus	252 (76.8)	652 (79.5)	1957 (76.2)	3471 (71.0)
				Reference
Intermediate	28 (8.5)	53 (6.5)	270 (10.5)	696 (14.2)
	0.6 (0.4, 0.8)	0.4 (0.3, 0.6)	0.8 (0.7, 0.9)	
	**0.0050**	**< 0.0001**	**< 0.0001**	
Inadequate	31 (9.5)	92 (11.2)	278 (10.8)	582 (11.9)
	0.7 (0.5 1.1)	0.9 (0.7, 1.1)	0.9 (0.8, 1.0)	
	0.1256	0.1864	0.0886	
**Insurance status at birth**				
Employer based insurance HMO or PPO	116 (35.4)	284 (34.6)	1011 (39.3)	1905 (39.0)
				Reference
Medi-Cal or public insurance	191 (58.2)	476 (58.1)	1377 (53.6)	2667 (54.5)
	1.2 (0.9, 1.5)	1.2 (1.0, 1.4)	1.0 (0.9, 1.1)	
	0.1962	**0.0391**	0.6629	
Self-pay	a	14 (1.7)	24 (0.9)	38 (0.8)
		2.1 (1.2, 3.5)	1.1 (0.7, 1.7)	
		**0.0077**	0.5937	
Other	17 (5.2)	46 (5.6)	158 (6.2)	281 (5.8)
	1.0 (0.6, 1.7)	1.1 (0.8, 1.5)	1.0 (0.9, 1.2)	
	0.9812	0.6107	0.6622	
**Prior PTB**				
Yes	34 (10.4)	63 (7.7)	72 (2.8)	76 (1.6)
	7.3 (4.8, 11.2)	5.3 (3.7, 7.4)	1.8 (1.3, 2.5)	
	**< 0.0001**	**< 0.0001**	**0.0003**	
No	294 (89.6)	757 (92.3)	2498 (97.2)	4815 (98.5)
				Reference
**Preeclampsia**				
Yes	122 (37.2)	267 (32.6)	421 (16.4)	304 (6.2)
	6.7 (5.3, 8.3)	4.3 (3.8, 5.0)	1.8 (1.6, 2.0)	
	**< 0.0001**	**< 0.0001**	**< 0.0001**	
No	206 (62.8)	553 (67.4)	2149 (83.6)	4587 (93.8)
				Reference
**Smoking during pregnancy**				
Yes	41 (12.5)	90 (11.0)	228 (8.9)	374 (7.7)
	1.7 (1.2, 2.3)	1.4 (1.1, 1.7)	1.1 (1.0, 1.3)	
	**0.0026**	**0.0029**	0.1352	
No	287 (87.5)	730 (89.0)	2342 (91.1)	4517 (92.4)
				Reference
**Body mass index [BMI (kg/m**^**2**^**)]**				
< 18.5	a	8 (1.0)	33 (1.3)	77 (1.6)
		0.8 (0.4, 1.5)	1.0 (0.7, 1.4)	
		0.4303	0.9879	
18.5 to < 25	58 (17.7)	147 (17.9)	438 (17.0)	1026 (21.0)
				Reference
25 to < 30	69 (21.0)	179 (21.8)	578 (22.5)	1172 (24.0)
	1.0 (0.7, 1.5)	1.1 (0.9, 1.3)	1.1 (1.0, 1.2)	
	0.8293	0.6170	0.1184	
30 to < 35	58 (17.7)	178 (21.7)	542 (21.1)	1023 (20.9)
	1.0 (0.7, 1.4)	1.2 (1.0, 1.5)	1.2 (1.0, 1.3)	
	0.9881	0.1323	**0.0228**	
35 to < 40	61 (18.6)	133 (16.2)	408 (15.9)	651 (13.3)
	1.6 (1.1, 2.3)	1.4 (1.1, 1.7)	1.3 (1.1, 1.4)	
	**0.0103**	**0.0114**	**0.0002**	
> 40	53 (16.2)	139 (17.0)	431 (16.8)	679 (13.9)
	1.4 (0.9, 2.0)	1.4 (1.1, 1.7)	1.3 (1.1, 1.5)	
	0.1114	**0.0101**	**0.0001**	

^a^not presented when *n* < 5

**Table 3:** Results of nativity*maternal characteristics and maternal characteristic*maternal characteristics interaction terms included during regression modeling, *p* < 0.1

Interaction terms by gestational group	***p***-value
**Early preterm**	
Nativity*Participation in WIC	0.0293
Preeclampsia*smoking	0.0008
Smoking*obesity	0.0004
**Late preterm**	
Nativity*Participation in WIC	0.0037
Preeclampsia*smoking	0.0318
Smoking*obesity	0.0081
**Early term**	
Nativity*Payer status at birth	0.0510
Smoking*obesity	0.0282

### Outcome variables – late PTB

Foreign-born status (cOR 0.6 (0.4, 0.7) and intermediate PNC utilization (cOR 0.4 (0.3, 0.6)) were significantly associated with a lower odds of late PTB. Prior PTB (cOR 5.3 (3.7, 7.4)), preeclampsia (cOR 4.3 (3.8, 5.0)), self-pay insurance status (cOR 2.1 (1.2, 3.5)), education < 12 years (cOR 1.4 (1.1, 1.7)), smoking (cOR 1.4 (1.1, 1.7)), class II obesity (cOR 1.4 (1.1, 1.7)), class III obesity (cOR 1.4 (1.1, 1.7)), and Medi-Cal (cOR 1.2 (1.0, 1.4)) were significantly associated with increased odds of late PTB. No significant associations were observed between late PTB and maternal age, and WIC participation (Table 2). Confounders between nativity and late PTB included maternal age. Significant interaction between nativity and late PTB, and between maternal characteristics included WIC participation*nativity (*p* = 0.0037), preeclampsia*smoking (*p* = 0.0318), and smoking*obesity (*p* = 0.0081) (Table 3).

### Outcome variables – early term birth

Nativity (cOR 0.7 (0.7, 0.8)) and intermediate PNC utilization (cOR 0.8 (0.7, 0.9)) were also significantly associated with lower odds of early term birth. Prior PTB (cOR 1.8 (1.3, 2.5)) and preeclampsia (cOR 1.8 (1.6, 2.0)) were significantly associated with increased odds of early term birth. In contrast to early PTB and late PTB, pre-pregnancy obesity across all three classifications, class I (cOR 1.2 (1.0, 1.3)), class II (cOR 1.3 (1.1, 1.4)), and class III (cOR 1.3 (1.1, 1.5), was significantly associated with increased odds of early term birth. No significant associations were observed between early term birth and maternal age, education, WIC participation, smoking, payer status at birth, or birth types (Table 2). Confounders between nativity and early term birth were not observed. Significant interactions between nativity and early term birth, and between maternal characteristics included smoking*obesity (*p* = 0.0282) (Table 3).

### Outcome variables – PTB subtype and nativity

The prevalence of total PTB, including subtypes spontaneous and indicated PTB, among all Black women with GDM was 13.3% (Table 4). Overall, 14.2% of US-born Black women with GDM had a PTB compared to 8.9% among foreign-born Black women. Study findings revealed significant differences in the proportion of total PTB by maternal country of origin (*p* <  0.0001), with the largest contribution towards PTB burden found among foreign-born Black women in California from Ghana (11.8%), Other (10.3), Nigeria (10.2%), Somalia (8.3%), and Ethiopia (6.9%), respectively (Table 4). There was no significant difference in PTB subtype based on maternal country of origin (*p* = 0.5913) in the sample population (Table 5).

**Table 4:** Prevalence of Total PTB, spontaneous PTB, and indicated PTB by maternal country of origin among Black women with GDM In California, 2013–2017, N = 8609

Country of origin	Number of mothers	Total PTB n (row%)	***p*** value
**All**	8609	1148 (13.3)	*p* < 0.0001
**U.S.**	7222	1024 (14.2)	
**Ethiopia**	346	24 (6.9)	
**Nigeria**	246	25 (10.2)	
**Somalia**	120	10 (8.3)	
**Eritrea**	91	a	
**Ghana**	68	8 (11.8)	
**Other**	516	53 (10.3)

**Table 5:** Prevalence of PTB by subtypes, PPROM, spontaneous labor with intact membranes, provider initiated, and unknown subtype, by maternal country of origin, among Black women with GDM in California, 2013–2017, *N* = 8609

Country of origin	PPROM n (row %)	Spontaneous labor with intact membranes n (row %)	Provider initiated n (row %)	Unknown subtype n (row %)	***p***-value
**U.S.**	235 (23.0)	430 (42.0)	333 (32.5)	26 (2.5)	0.5913
**Ethiopia**	a	7 (29.2)	13 (54.2)	a	
**Nigeria**	a	10 (40.0)	12 (48.0)	a	
**Somalia**	a	a	a	a	
**Eritrea**	a	a	a	a	
**Ghana**	a	a	a	a	
**Other**	10 (18.9)	22 (41.5)	18 (34.0)	a	

### Outcome variables – PTB risk after adjustment for confounding and interaction analysis

After stepwise backward regression analyses, covariates and interaction terms in the final model for early PTB included maternal age < 24, preeclampsia, smoking, obesity, prior PTB, interaction of preeclampsia*smoking, and interaction of smoking*obesity. Covariates and interactions terms that remained in the final model for late PTB included education < 12 years, preeclampsia, smoking, obesity, prior PTB, and the interaction of smoking*obesity.

Covariates and interaction terms that remained in the final model for early term birth included preeclampsia, prior PTB, and obesity. We found a protective association of foreign-born status for early PTB (aOR 0.56 (0.38, 0.82)), late PTB (aOR 0.57 (0.45, 0.73)) and early term birth (aOR 0.67 (0.58, 0.77)) (Table 6). Foreign-born status had no differentiating impact on PTB severity among Black women with GDM, given the similar aOR with overlapping CIs for early PTB and late PTB. Similarly, foreign-born status had no differentiating impact between PTB and early term birth.

**Table 6:** Fully adjusted associations for PTB, by severity, and early term birth based on immigrant status, *N* = 8609

	Early preterm	Late preterm	Early term
	aOR (95% CI)^**a**^	aOR (95% CI)^**b**^	aOR (95% CI)^**c**^
Nativity	0.56 (0.38, 0.82)	0.57 (0.45, 0.73)	0.67 (0.58, 0.77)

^a^Included in the final model: maternal age < 24, preeclampsia, smoking, obesity, prior preterm birth, interaction of preeclampsia*smoking, interaction of smoking*obesity

^b^Included in the final model: education < 12 years, preeclampsia, prior preterm birth, smoking, obesity, smoking*obesity

^c^Included in the final model: preeclampsia, obesity, prior preterm birth

## Discussion

Among the population of Black women with GDM in California, foreign-born status remained protective against PTB, irrespective of severity, subtype, and maternal country of origin, and early term birth. Our data further support the occurrence and analytic of excess PTB among U.S.-born Black women with GDM.

Our data suggest that PTB among multiparous Black women with GDM in California may not be a transmittable genetic defect, obligating U.S.-born Black women and their children to a lifetime of reproducing harm as a result of birthing while Black. Our findings also suggest the need to conduct additional social and clinical epidemiological studies using patient reported outcome measures and patient reported experience measures of PTB to examine the social, structural, and clinical determinants of PTB, by severity and subtype, among Black women with GDM in California. Social and clinical epidemiological studies can be strengthened with the reporting of patient measures defined and developed for, by, and with Black birthing communities so that clinicians and Black birthing communities can make evidence informed decisions together to improve patient centered and focused care.

Our study findings also demonstrate that after a history of a prior PTB, preeclampsia was the overall greatest predictor of early PTB, late PTB, and early term birth among Black women with GDM. The large effect of preeclampsia risk for all gestational groups, given no evidence of interaction between nativity and preeclampsia, suggests a potential role for unmeasured independent variables, such as stress, inflammation, or chronic hypertension as potential pathways for PTB [34, 52]. GDM and preeclampsia may also act synergistically through a common pathway of inflammation, where PTB may also be an indicator of vascular dysfunction [53], given the significant associations between, preeclampsia, smoking and PTB. The interaction analyses in our study showed a significant interaction between smoking and obesity across three gestational groups, perhaps suggesting shared pathophysiologic mechanisms leading to inflammation and vasoconstriction among Black women with GDM, resulting in a phenomenon of births prior to 39 weeks’ gestation [54]. Thus, our data support the treatment of aspirin among Black women with GDM for the purposes of primary prevention of preeclampsia, particularly early-onset preeclampsia, which could potentially prevent indicated or spontaneous PTB or early term birth, by delaying the gestational age at birth with preeclampsia [55].

These data underscore the need for more culturally rigorous [56], theoretically driven and relevant epidemiologic studies and health services research to better identify and characterize modifiers, mediators and confounders of the association between nativity, racism, and PTB among Black women with GDM. Better data has the potential to lead to improved screening protocols and hopefully improved triage, management, and allocation of clinical and social resources.

Predictors for PTB, by severity, and early term births were different in the three gestational groups, based on nativity, as previously described in the results about regression modeling for each gestational group. After adjusting for these predictors, the association of nativity with early PTB and late PTB, respectively, remained protective (aOR = 0.56 and aOR = 0.57), suggesting that these predictors did not fully explain the protective effect of foreign-born status among Black women with GDM in California. In addition, the association of nativity with early term births remained similar (aOR = 0.73), providing further evidence that these predictors did not fully explain the protective effect of foreign-born status. Thus, the disparity in early term births between U.S.-born and foreign-born Black mothers with GDM based on nativity raises questions and concerns about potential variations in quality of care, patient understanding and autonomy, and glycemic surveillance, reporting, and control mechanisms. As such, further investigation of structural, social, and clinical factors will be critical areas of investigation in follow-up studies.

### Clinical implications

In this study, the most common predictors of PTB among Black women with GDM, regardless of PTB severity, were history of prior PTB, preeclampsia, smoking, and class II obesity. Given the context of structural inequities in perinatal and reproductive health in the U.S., and the American College of Obstetricians & Gynecologists (ACOG) 2013 treatment guidelines for preeclampsia with severe features at > 34 weeks versus < 34 weeks, [57], where the recommendation for pregnancy continuation occurs only at facilities with maternal and neonatal intensive care resources, preeclampsia, particularly early-onset, is the most likely reason for provider initiated PTB and early term birth among U.S. born Black women with GDM. Provider initiated births likely result in prolonged use of oxytocin for induction and augmentation of labor, as well as prolonged exposure to magnesium sulfate for the purposes of seizure prophylaxis or fetal neuroprotection. Obesity [58], prolonged use of oxytocin [59], and magnesium sulfate all independently and likely synergistically increase the risk of uterine atony leading to increased risk of postpartum hemorrhage (PPH) requiring blood transfusion.

Additionally, a subanalysis of birth types by gestational groups revealed high hospital performance of cesarean births among 3 out of 4 Black women with GDM who experienced an early PTB, and nearly every 2 out of 3 Black women with GDM who experienced a late PTB in our sample of Black women across California. Given the most common predictors of PTB among Black women with GDM, regardless of PTB severity, were preeclampsia, smoking, and class II obesity, within the context of provider-initiated births as a result of preeclampsia, and the high hospital performance of early PT and late PT cesarean births, our data raises concern for the increased burden of PPH and its social and clinical sequelae among Black women with GDM in California who experience PTB. A potentially prolonged labor induction with prolonged use of oxytocin and magnesium sulfate, along with the inability to use methergine as a primary uterotonic due to its contraindication in patients with hypertensive disease, increases the likelihood that Black women with GDM, obesity, and preeclampsia will likely experience uterine atony with PPH, requiring administration of blood products, particularly during a cesarean birth. These data suggest a need for different clinical approaches to optimizing the care, experiences, and outcomes for Black women with GDM and obesity, who either present and/or develop preeclampsia at early and late PTB, given the risk of both short- and long-term perinatal morbidity that may impact the woman and child.

Additionally, hemorrhage that leads to blood transfusion is a leading cause of severe maternal morbidity (SMM) [60] and Black women are more likely to experience a PPH [61]. These data suggest a need to further examine the relationship between blood transfusion, cesarean birth, and PTB. PPH and blood transfusion are not considered predictors of PTB, but more so, they serve as indications of SMM with PTB. A population-based study in California that identified the correlates of dual burden of SMM and PTB concluded that the strongest predictors of dual burden of PTB and SMM were hypertensive disorders with preeclampsia and multiparous primary cesarean [62]. Thus, our findings suggest a replication of the study by Lyndon et al. [62] to more appropriately examine the associations between blood transfusion, PTB, and SMM among Black women with GDM.

### Strengths and limitations

To the best of our knowledge, as of April 2020, this is the first quantitative population health research study to evaluate the association of nativity, PTB, by severity and subtype, and early term birth, respectively, in a high-risk population of Black women with GDM in California. Other study strengths include: 1) use of a large, population-based sample with a single racial group exclusive to Black women with GDM; 2) sufficient statistical power to adjust for important covariates; 3) delineation between PTB subtypes: PPROM, spontaneous PTB with intact membranes, provider initiated PTBs, and unknown subtype; 4) stratification of PTB subtypes by maternal country of origin for the countries with the largest proportion of foreign-born Black women with GDM in the sample; 5) evaluation of interactions between nativity and all maternal characteristics, and between key maternal characteristics, including preeclampsia, smoking, and obesity; and 6) explicit acknowledgement and utilization of an anti-racist praxis undergirding our study assumptions and methodologies.

While we believe that the strengths of the study far outweigh its weaknesses, it is important to acknowledge a number of limitations that will be critical to address in future studies. These limitations include: 1) an inability to diagnose or find a rationale for medically-indicated early term birth; 2) a lack of information available for us to determine and assess patient, provider and systems level factors such as patient knowledge and autonomy, nutritional intake, referrals to paraprofessionals, magnitude of glycemic control, and group GDM and prenatal support; and 3) inability to measure key variables associated with birth and nativity such as pregnancy wantedness, stressors, and supports within the context of the social ecological, life course, and resiliency perspectives, and access to social capital.

## Conclusions

There is an urgent need for advanced epidemiological and health services research to identify the underlying causes of excess PTB and early term births among U.S.-born Black women with GDM. This study serves as a building block for epidemiologists, clinicians, and clinician scientists, and suggests that PTB and early term birth may be in part due to chronic inflammation or vasculature dysfunction in response to chronic life stressors. New, prospective, and longitudinal research would benefit from a focus on measures of acculturation, life course experience of structural and interpersonal gendered racism and other stressors, biomarkers, and glycemic control in order to gain a fuller understanding of PTB and early term birth drivers. In addition, given the strong associations between PTB, GDM, preeclampsia, and obesity, future studies should examine the relationships between glycemic control intensity and type of medical management for GDM, PTB and early term birth subtypes, including spontaneous and clinically indicated PTB, and development of preeclampsia in U.S - and foreign-born Black women with GDM.

Additional studies are needed to explore the interactions between preeclampsia, smoking, obesity, and life stressors and their effect on birth outcomes. Our findings support the need for randomized trials to assess pharmacologic interventions such as baby aspirin for the prevention and reduction of inflammation among women with a history of early onset preeclampsia and prior PTB less than 34 completed gestational weeks, history of early onset preeclampsia, and prior preterm birth less than 34 completed gestational weeks, particularly among Black women with any diabetes, aged > 35 years or older, obese, and other social and clinical co-morbidities. Our data also support evidence-based socio-clinical interventions such as community informed, Midwifery-led, racially concordant group prenatal and postpartum care with wraparound Doula support services [29], in collaboration with Obstetricians for Black women, particularly for U.S.-born Black women whose pregnancies are impacted by life stressors, gendered racism, GDM, preeclampsia, obesity, smoking, and other significant personal and family history.

## Data Availability

The data that support the findings of this study are available from The California Department of Public Health, but restrictions apply to the availability of these data, which were used under license for the current study, and so are not publicly available. Data are however available from the authors upon reasonable request and with permission from the California Department of Public Health.

## Abbreviations

BMI: Body mass index
GDM: Gestational diabetes mellitus
Medi-Cal: California’s Medicaid Program
PNC: Prenatal care
PTB: Preterm birth
PPROM: Preterm premature rupture of membranes
PPH: Postpartum hemorrhage
WIC: Women, infant, and children’s program
